# Engineering the future of 3D pathology

**DOI:** 10.1002/cjp2.347

**Published:** 2023-11-02

**Authors:** Jonathan TC Liu, Sarah SL Chow, Richard Colling, Michelle R Downes, Xavier Farré, Peter Humphrey, Andrew Janowczyk, Tuomas Mirtti, Clare Verrill, Inti Zlobec, Lawrence D True

**Affiliations:** ^1^ Department of Mechanical Engineering University of Washington Seattle WA USA; ^2^ Department of Laboratory Medicine & Pathology University of Washington School of Medicine Seattle USA; ^3^ Department of Bioengineering University of Washington Seattle USA; ^4^ John Radcliffe Hospital University of Oxford Oxford UK; ^5^ Sunnybrook Health Sciences Centre University of Toronto Toronto Canada; ^6^ Public Health Agency of Catalonia Lleida Spain; ^7^ Department of Urology Yale School of Medicine New Haven CT USA; ^8^ Wallace H Coulter Department of Biomedical Engineering Emory University and Georgia Institute of Technology Atlanta GA USA; ^9^ Geneva University Hospitals Geneva Switzerland; ^10^ Helsinki University Hospital and University of Helsinki Helsinki Finland; ^11^ Emory University School of Medicine Atlanta GA USA; ^12^ NIHR Oxford Biomedical Research Centre Oxford University Hospitals NHS Foundation Trust Oxford UK; ^13^ Institute for Tissue Medicine and Pathology University of Bern Bern Switzerland

**Keywords:** light‐sheet microscopy, nondestructive 3D pathology, digital pathology, prostate cancer, prognosis

## Abstract

In recent years, technological advances in tissue preparation, high‐throughput volumetric microscopy, and computational infrastructure have enabled rapid developments in nondestructive 3D pathology, in which high‐resolution histologic datasets are obtained from thick tissue specimens, such as whole biopsies, without the need for physical sectioning onto glass slides. While 3D pathology generates massive datasets that are attractive for automated computational analysis, there is also a desire to use 3D pathology to improve the visual assessment of tissue histology. In this perspective, we discuss and provide examples of potential advantages of 3D pathology for the visual assessment of clinical specimens and the challenges of dealing with large 3D datasets (of individual or multiple specimens) that pathologists have not been trained to interpret. We discuss the need for artificial intelligence triaging algorithms and explainable analysis methods to assist pathologists or other domain experts in the interpretation of these novel, often complex, large datasets.

## Introduction to nondestructive 3D pathology

Interest in nondestructive 3D pathology, wherein large tissues samples are imaged laterally and in depth, has grown in recent years as a complement to traditional slide‐based 2D pathology [1, 2]. This has been facilitated by advances in high‐throughput optical imaging techniques, including light‐sheet microscopy [1, 2, 3, 4, 5], optical coherence tomography [6, 7], photoacoustic microscopy [8, 9, 10], and micro‐computed tomography (μCT) [11, 12, 13, 14]. In addition, tissue‐clearing approaches that render thick tissues transparent for optical imaging [15, 16, 17, 18, 19], and advanced computational tools, have catalyzed further growth in 3D pathology. These computational tools include machine‐learning analysis methods [1, 20, 21, 22, 23, 24, 25, 26, 27, 28] and virtual staining methods to enable tissues, imaged with different modalities, to mimic the appearance of H&E histology [9, 29, 30, 31, 32, 33] as it continues to be the gold standard for most diagnostic determinations. Slide‐free 3D pathology offers several technical advantages when compared with traditional 2D pathology: (1) improved image sampling of large volumes of tissue rather than sparse imaging of those volumes with standard thin tissue sections; (2) volumetric imaging of diagnostically relevant 3D structures and extraction of novel 3D spatial biomarkers; and (3) nondestructive imaging, which allows intact tissue specimens (e.g. core‐needle biopsies) to be made fully available for downstream molecular assays. Ultimately, at the heart of 3D pathology is the hypothesis that nondestructive volumetric microscopy of tissue specimens can improve accuracy in diagnosis, prognostication, and prediction of treatment response.

Most clinically motivated studies in 3D pathology have thus far focused on characterizing and quantifying diseased tissues, including different stages and grades of disease progression [34, 35, 36, 37], rather than trying to improve our understanding of benign tissue anatomy. For example, recent studies have shown that quantifying the morphology of prostate glands and nuclei in 3D versus 2D allows for improved classification of aggressive versus indolent tumors [26, 27]. Likewise, 3D microscopy has been used for morphometric characterization of human livers as a function of different stages of nonalcoholic fatty liver disease, showing that there are cellular and tissue signatures (e.g. bile canaliculi) that correlate with disease progression [37]. Other studies in 3D pathology have provided insights that were previously ambiguous with 2D pathology methods. For example, recent studies on the 3D microstructure of pancreatic ductal adenocarcinoma have shown that tumor infiltration occurs along collagen fibers aligned with tissue structures such as vessels, nerves, and other glands [36]. In a study on colorectal cancer, 3D pathology revealed that tertiary lymphoid structures are often interconnected rather than isolated as they often appear in 2D histology [34]. This same study showed that structures that appear as isolated tumor buds in 2D are often extensions of larger tumor masses when viewed in 3D.

## Advantages of 3D pathology

**Figure 1:**
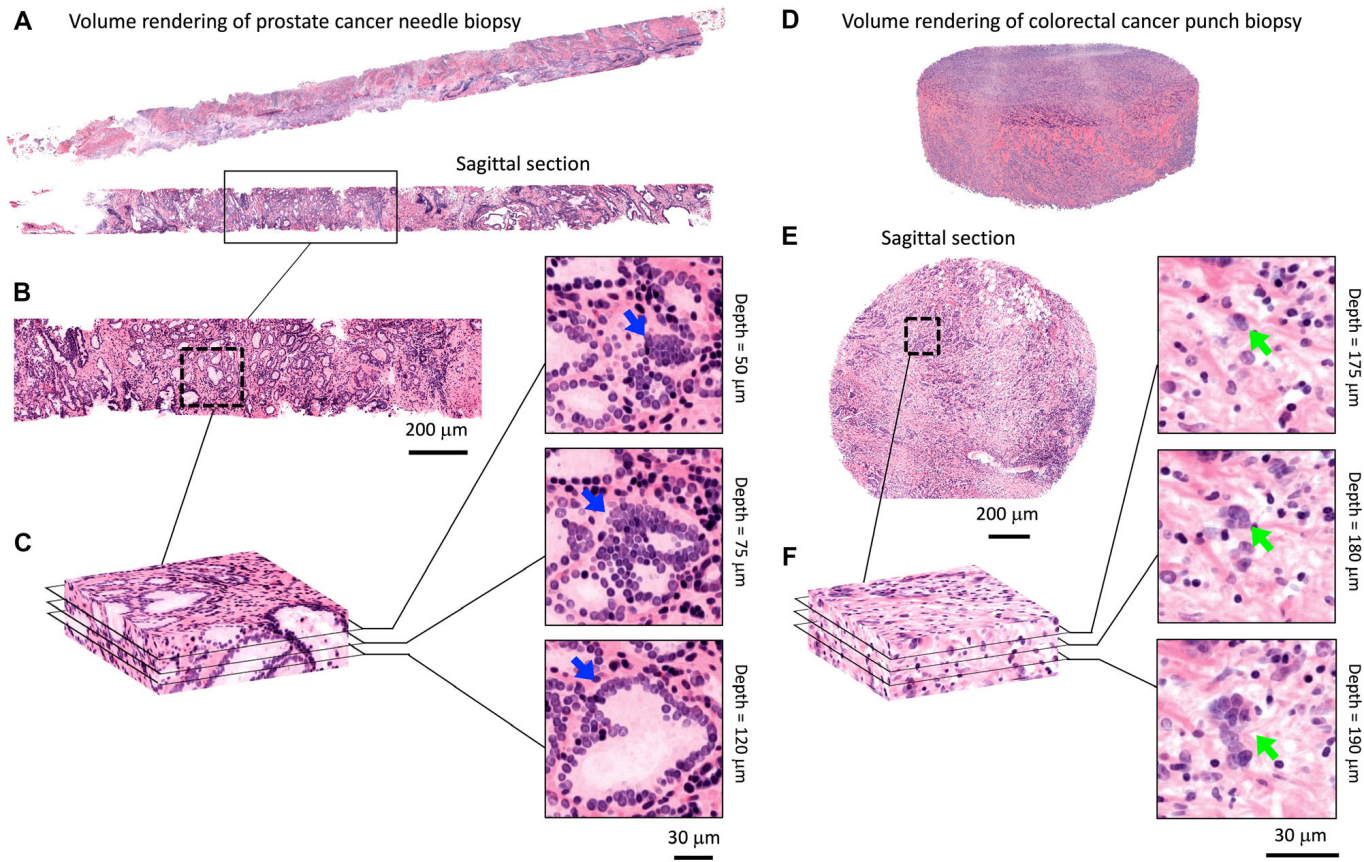
Examples of 3D pathology datasets with diagnostically significant spatial variations. These clinical specimens (FFPE) were deparaffinized, stained with a fluorescent analog of H&E, optically cleared to make them transparent, and imaged with OTLS microscopy. The volumetric fluorescence datasets were then false colored to mimic the appearance of standard H&E staining. (A) Volume rendering of a prostate core‐needle biopsy specimen. (B) A cropped view of one sagittal section from the prostate biopsy. (C) A zoomed‐in view of a volumetric block of data where a series of computationally generated 2D cross sections is shown at different depths. Here, a cancer gland appears poorly formed (Gleason pattern 4) at one depth but is revealed to be a tangential section of a fully formed gland (Gleason pattern 3) upon inspection of adjacent depths (blue arrows). (D) Volume rendering of a 3‐mm diameter punch biopsy of colorectal cancer. (E) A sagittal section is shown. (F) A zoomed‐in view of a volumetric block of data where a series of 2D cross sections is shown at different depths. Here, a few tumor cells appear to be clustered as an isolated ‘tumor bud’ (<4 cells) but are revealed to be connected to a larger mass of tumor cells upon inspection of adjacent depths (green arrows). 3D renderings created with the assistance of Imaris software.

For clinical diagnostic applications, there are at least four broad categories in which 3D pathology has the potential to provide value compared with 2D tissue sections: (1) more accurate characterization of convoluted and/or infiltrative microstructures, such as the observation of poorly formed prostate glands in 2D sections (Gleason pattern 4), which may be revealed as tangential sections of fully formed glands in 3D (Gleason pattern 3); or the observation of tumor buds in 2D sections, which may be revealed as infiltrative extensions of a larger tumor mass in 3D [38] (Figure 1). (2) More accurate characterization of complex and heterogeneous distributions of cells and their interactions, such as the tumor‐immune microenvironment, in which a patient's response to an immunotherapy can depend upon how well immune cells are able to migrate into a tumor mass (‘hot tumors’) versus being sequestered at the periphery of the tumor (‘cold tumors’). Since the infiltrative margins of many tumors are highly heterogeneous and spatially irregular, it can be difficult to accurately quantify these tumor‐immune cell interactions by viewing a few thin 2D sections [34, 39]. (3) The detection and quantification of diagnostically significant tissue features that are somewhat rare on 2D sections, such as lymphovascular invasion, perineural invasion, minimal residual disease, and tertiary lymphoid structures, but which are more prevalent in large 3D pathology datasets [34]. The ability to identify and quantify the presence of such objects with higher precision and statistical power than current binary reporting schemes would be of obvious value. (4) More sensitive and comprehensive assessment of disease invasion, such as near the surgical margins (e.g. extraprostatic extension), which can affect patient‐management decisions.

## Challenges with visual interpretation of 3D pathology

A major challenge with 3D pathology for human observers (i.e. pathologists) is that it provides orders of magnitude more data for a workforce that is already overburdened. Our experience is that large 3D pathology datasets can potentially yield greater interpathologist disagreement since each pathologist may view different regions of a large dataset. For example, in a preliminary study involving 161 prostate biopsies, we asked a panel of six pathologists (coauthors of this perspective article) to perform Gleason grading based on viewing depth stacks of 3D pathology data versus viewing a few 2D levels per biopsy. The median weighted Cohen's kappa value for 3D pathology was 0.48, which was slightly worse (in terms of interobserver agreement) than the kappa value of 0.53 for 2D pathology. Second, standard workflows and guidelines for the interpretation of 3D pathology datasets have yet to be established, unlike with 2D tissue sections. Concepts relied upon by pathologists for the interpretation of 2D sections may not be easily extrapolated to 3D. As mentioned previously, certain structures of prognostic significance, such as poorly formed glands in prostate tissues, or tumor buds, can often be artifacts of viewing 2D cross sections of a 3D object. Furthermore, certain artifacts introduced by the preparation of formalin‐fixed paraffin‐embedded (FFPE) sections, such as retraction artifacts (due to dehydration), have become diagnostically useful to pathologists, but may no longer be present in 3D pathology datasets that are acquired nondestructively (slide free). On the other hand, virtual staining techniques have the advantage of generating ‘H&E‐mimicking’ images that are much more consistent (in color space) compared with standard H&E‐stained slides [30], which can be advantageous for pathologists. A final challenge is that the method by which 3D pathology datasets should be visualized is not clear. In some cases, a sequential stack of images as a function of depth may be most informative, but in other cases, a volumetric reconstruction may be superior [40, 41, 42]. The ideal visualization software should at the very least allow pathologists to rotate specimens so that they may be observed from various perspectives and to view various cross‐sectional locations (i.e. different depths), which collectively could help to resolve certain ambiguities that are inherent to 2D histology.

## The value of artificial intelligence

Because of the size and complexity of 3D pathology datasets, there is a need for computational tools [i.e. machine learning, often referred to as ‘artificial intelligence’ (AI)] to facilitate analysis. A low‐risk application of AI would be to triage the massive datasets generated by 3D pathology so that pathologists can focus their attentions on the most diagnostically important 3D subregions or 2D levels (cross sections) [22, 25]. Ideally, with AI‐triaged 3D pathology, it will be possible to improve diagnostic sensitivity/accuracy by imaging larger amounts of tissue, while also reducing pathologist workloads – a compelling value proposition to drive clinical adoption. A recent study demonstrated this concept of AI‐triaged 3D pathology for improving the detection of neoplasia in endoscopic biopsies from patients with Barrett's esophagus [25]. On the other end of the spectrum, fully computational analysis of 3D samples, including with black‐box deep learning (DL) networks [24, 28], would need to be validated in large prospective studies to elucidate their potential novel value for decision‐making in patient care. However, for pathologists seeking spatial and molecular insights, there would be great value in explainable analysis methods, such as with machine classifiers based on ‘hand‐crafted’ features or *post hoc* examination of DL models and their attention maps [20, 23, 24, 25, 26, 27]. The field of explainable AI is still under rapid development and will hopefully play an important role in revealing novel biological insights and 3D spatial biomarkers of disease progression, prognosis, and treatment response.

As a technical comment, most existing computational pathology methods utilize 2D inputs. While these AI methods do not take advantage of 3D features, we can still expect an improvement in performance due to the vastly greater amount of 2D imaging data that is provided in a 3D pathology dataset (i.e. improved sampling). If we train AI models to also incorporate/embed 3D features from 3D image chunks (versus 2D patches), we should expect an additional performance boost. This has been demonstrated in a recent article that analyzed 3D pathology data acquired with both open‐top light‐sheet (OTLS) microscopy and μCT [15]. More specifically, it was shown that DL analysis based on 3D chunks is superior to analysis of the same tissue volumes in a slice‐by‐slice manner (i.e. 2.5D analysis), and that both of these approaches are superior to the analysis of a single 2D slice from each tissue volume.

## Summary and outlook

In summary, we envision a future in which clinicians can benefit from the wealth of data and feature‐rich spatial (and molecular) insights that 3D pathology can offer, as a complement to traditional 2D histology. Pathologists and researchers in many fields, from developmental biologists to tissue engineers, would benefit from 3D pathology datasets of both benign and diseased organs, along with standardized anatomic terminologies and quantitative measurements based on such datasets [36, 43]. Computational tools are necessary to provide insights on how best to interpret novel 3D datasets, and to triage these large datasets such that interpretation by pathologists is efficient. In some cases, automated diagnoses may be possible with AI tools if pathologists are involved in training, validation, and continuous oversight. However, 3D pathology‐derived features, like all novel biomarkers, require vigorous validation through prospective studies if intended to change patient‐level decision making. There are opportunities and potential advantages for the integration of 3D pathology with other ‘omics techniques, such as genomics and radiomics, especially since the nondestructive nature of 3D pathology makes more tissue available for downstream molecular assays, and the large volumes of tissue that are imaged with 3D pathology can be more easily co‐registered with radiology datasets for multimodal analyses. There are also potential opportunities to achieve 3D pathology in an *in vivo*/*in situ* setting with miniature and endoscopic microscopy devices [44]. Pathologists are critically needed to facilitate these transformations in healthcare, but the pathologists of the future will need additional skillsets, most notably in the data sciences.

## Author contributions statement

JTCL contributed to conceptualization, formal analysis, supervision, visualizations, funding acquisition, investigation, methodology, writing original manuscript and project administration. SSLC contributed to conceptualization, data collection, formal analysis, visualization, investigation, methodology, writing original manuscript and project administration. MRD, RC, XF, PH, AJ, TM, CV, IZ and LDT contributed to investigation, formal analysis and funding acquisition. All authors contributed to reviewing and editing the manuscript.

## Disclaimer

Any opinions, ifindings, and conclusions or recommendations expressed in this material are those of the authors and do not necessarily reflect the views of the US National Institutes of Health (NIH), National Science Foundation (NSF), Prostate Cancer United Kingdom (PCUK) charity, US Department of Defense (DoD), Oxford University Hospitals NHS Foundation Trust, the UK National Institute for Health Research (NIHR), the Department of Health, United Kingdom, or the United States government.
